# Contributions of Composition and Interactions to Bacterial Respiration Are Reliant on the Phylogenetic Similarity of the Measured Community

**DOI:** 10.1007/s00248-017-0982-2

**Published:** 2017-04-27

**Authors:** Damian W. Rivett, Andrew K. Lilley, Gary J. Connett, Mary P. Carroll, Julian P. Legg, Kenneth D. Bruce

**Affiliations:** 1 Institute of Pharmaceutical Science, King’s College London, Franklin-Wilkins Building, London, UK; 2 Present Address: Division of Ecology and Evolution, Imperial College London, Silwood Park Campus, Ascot, UK; 3 UK National Institute for Health Research, Southampton Respiratory Biomedical Research Unit, University Hospital Southampton NHS Foundation Trust, Southampton, UK

**Keywords:** *Pseudomonas aeruginosa*, Niche radiation, Biodiversity-ecosystem function, Intraspecific variation, Bacterial consortia, Carbon utilisation

## Abstract

Bacterial diversity underpins many ecosystem functions; however, the impact of within-species variation on the relationship between diversity and function remains unclear. Processes involving strain differentiation, such as niche radiation, are often overlooked in studies that focus on phylogenetic variation. This study used bacterial isolates assembled in two comparable microcosm experiments to test how species variation affected ecosystem function. We compared the relationship between diversity and activity (CO_2_ production) in increasingly diverse multispecies microcosms and with multiple ecotypes of a single species. The bacteria used were isolated from a low-diversity environment and are species of potential clinical significance such as *Pseudomonas aeruginosa*. All isolates were profiled for single carbon source utilisation. These data showed an increased breadth of resource use in the multiple ecotypes when compared to the mixed-species. The study observed significantly increasing respiration in more complex mixed-species assemblages, which was not observed when ecotypes of a single species were combined. We further demonstrate that the variation observed in the bacterial activity was due to the roles of each of the constituent isolates; between different species, the interactions between the isolates drove the variation in activity, whilst in single species, assemblage variation was due to which isolates were present. We conclude that both between- and within-species variations play different roles in community function, although through different mechanisms, and should be included in models of changing diversity and ecosystem functioning.

**Electronic supplementary material:**

The online version of this article (doi:10.1007/s00248-017-0982-2) contains supplementary material, which is available to authorized users.

## Introduction

Whilst microbial communities are often highly phylogenetically and functionally diverse, much of the variation in these communities is masked at the sub-species level [1]. Intraspecific variation though is important and forms one mechanism by which single species can come to proliferate in environments, for example by radiating specialists into a broad range of niches thereby reducing competition and increasing complementary interactions [2–5]. Many current studies infer specific functions to individual species within communities as extrapolated from high throughput sequencing data, e.g. ([6]). Addressing the impact of intraspecific interaction and variation is, however, extremely challenging using such approaches. Manipulative studies, which give researchers the power to create experimental bacterial consortia, offer important alternative approaches through which ecosystem function can be related to changes in diversity [7]. Such biodiversity-ecosystem function (BEF) experiments have been reported in which bacterial species have been selected on morphological, phylogenetic or functional criteria [7–10]. Here, we selected bacteria based on data from both 16S rRNA gene sequence and single carbon source usage to set up two concurrent BEF experiments. The first BEF experiment analysed between-species interactions among phylogenetically diverse species, and the second studied within-species interactions between phylogenetically indistinguishable yet functionally diverse ecotypes [1] of the same species. This approach allowed us to conduct an investigation into how the “unseen” variation within a species can affect ecosystem functioning.

Bacteria were isolated from expectorated sputum sampled from individuals with cystic fibrosis (CF). CF respiratory infections are typically chronic by adulthood, with relatively low bacterial species diversity, high bacterial counts and varied habitats. In these conditions, BEF experiments are especially applicable [11–13]. This environment has also been observed to enable the adaptive radiation of the clinically important bacterial species *Pseudomonas aeruginosa* [4]. A total of 23 isolates were chosen of which 12 were identified as *P. aeruginosa.* The remaining 11 identified as a species from one of nine commonly isolated genera that naturally co-occur, representing the abundance of these genera observed in the sputum samples used (Table S1). Microcosms were assembled as described by the random partitions design [14]; within each set of mixed-species and mixed-ecotype combinations, a total of 28 microcosms were created containing either 1, 2, 3, 4, 6 or 12 isolates, with each isolate present once at each richness level. Two pools of isolates were used in this study, one using a mixture of 12 bacterial species and the other using 12 phylogenetically indistinguishable *P. aeruginosa* ecotypes (one *P. aeruginosa* isolate was used in both pools). These bacterial combinations were grown for 24 h at 37 °C statically in 30 mg mL^−1^ tryptone water. Respiration was measured using the MicroResp™ system and abundances of each isolate in the mixed-species microcosms were monitored through 16S rRNA gene terminal restriction fragment (T-RF) profiling from DNA extracted from the microcosms [15] (see Supplementary Methods).

**Fig. 1:**
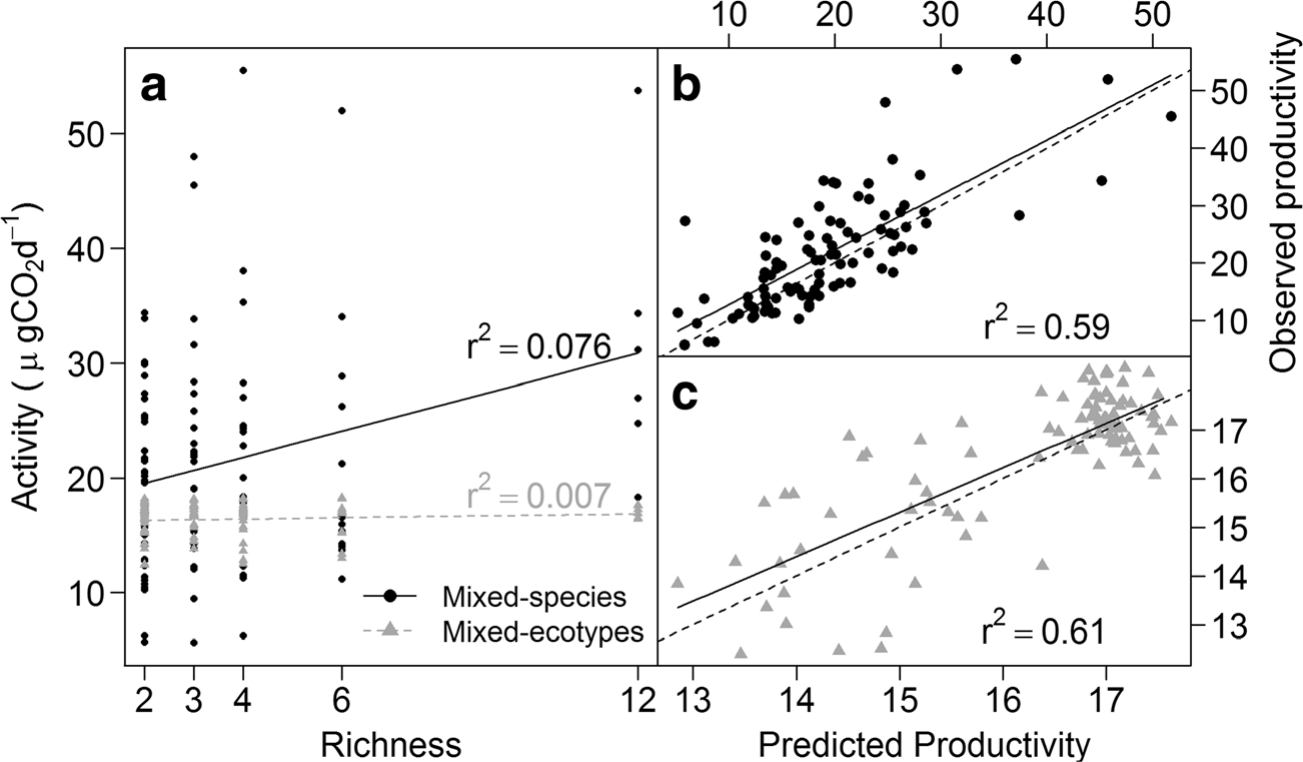
Bacterial respiration is dependent on increasing phylogenetic richness rather than ecotypic richness. **a** A direct comparison of activity (μg CO_2_ day^−1^) of the microcosms with mixed-species ((*black circle*) *black*, *solid line*) and mixed-ecotypes only ((*grey triangle*) *grey*, *dotted line*). Community activity was shown to increase as more species were added to a microcosm; however, this effect was not observed as the number of *P. aeruginosa* ecotypes increased. This observed activity was shown to be predicted by the monocultures (*solid black lines* represent the trend of the data with the *dotted black lines* showing the 1:1 relationship for comparison) when considering both **b** mixed-species, in which the relative abundances were included and **c** mixed-ecotype microcosms, where activity was divided equally between the isolates

We observed (Fig. 1a) an increased respiration by 1.13 μg CO_2_ day^−1^ species^−1^ (*F*_1,10_ = 8.77, *P* = 0.01) with increasing richness in the mixed-species BEF experiment. In contrast, in the mixed-ecotype experiment using Pseudomonads, there was no significant trend (0.04 μg CO_2_ day^−1^ ecotype^−1^, *F*_1,10_ = 1.35, *P* = 0.27). Whilst no significant differences (*t*_71_ = 1.40, *P* = 0.16) were observed between the mean monoculture activity values (±1 standard deviation throughout) of the mixed-species (17.53 ± 7.68 μg CO_2_ day^−1^) and *P. aeruginosa* ecotypes (16.24 ± 1.46 μg CO_2_ day^−1^), the mixed-species assemblages were consistently more active, and varied, than mixed-ecotype microcosms containing two or more isolates. This suggested that the positive trends observed in this mixed-species BEF experiment could be due to complementarity (niche differences or synergistic interactions) and as such not solely through selection effects [5].

Further investigation of the data (Table 1) using a general linear model [14] found that the effect of bacterial interactions was both significant, greater significance was observed between mixed-species (*F*_4,10_ = 15.91, *P* < 0.01) than mixed-ecotype assemblages (*F*_4,10_ = 4.40, *P* = 0.02). We investigated this further by plotting the observed against the predicted activity for both BEF experiments (Fig. 1b, c) [10]. Here, the null model was equivalence between the observed and predicted values (1:1 relationship) with predicted values calculated using the total constituent monoculture activity divided by the number of species within an assemblage. We found that in the mixed-species assemblages (Fig. 1b), a significant difference between the prediction and the null model (*β* = 0.77, *t*_282_ = 2.03, *P* = 0.043) was observed. This was eliminated when the relative abundances (T-RF profiles) were added into the predictions (*β* = 0.96, *t*_282_ = 1.53, *P* = 0.127), thereby accounting for relative differences in species abundance [10]. Interactions between bacterial species are manifested by changes in their relative abundances [9], by both positive (e.g. cross-feeding [2]) and negative (e.g. competitive exclusion [16]) mechanisms. The prediction got closer to the null hypothesis with the inclusion of the interactions. This is not dependent solely on diversity but on the interplay between the species. By contrast, in *P. aeruginosa* ecotype assemblages, the null model prediction (Fig. 1c) was not significantly different from the null model (*β* = 0.91, *t*_282_ = 0.07, *P* = 0.206), thereby suggesting that any differences in ecotype abundance did not impact upon overall activity.

**Table 1:** Linear models estimating the effect of the additive model, composition and interactions on bacterial respiration. These biological factors were analysed with respect to the partitioned species pool and microcosm variance in the mixed-species and *P. aeruginosa* ecotype microcosms [14]. Residual degrees of freedom (Res.df) are those remaining after a variable has been added sequentially to the model

Step	Variable	Res.df	*F*	*P*
Multispecies microcosms				
1	Respiration ~1	167	–	–
2	1+ additive model	166	7.31	0.02
3	2+ composition	154	1.1	0.38
4	3+ interactions	150	15.91	<0.01
5	4+ partitioned species pool	138	3.14	<0.01
6	5+ microcosm variation	83	1.91	<0.01
*P. aeruginosa* microcosms				
1	Respiration ~1	167	–	–
2	1+ additive model	166	0.14	0.71
3	2+ composition	154	1.93	0.05
4	3+ interactions	150	4.4	0.02
5	4+ partitioned species pool	138	20.66	<0.01
6	5+ microcosm variation	83	1.19	0.26

**Fig. 2:**
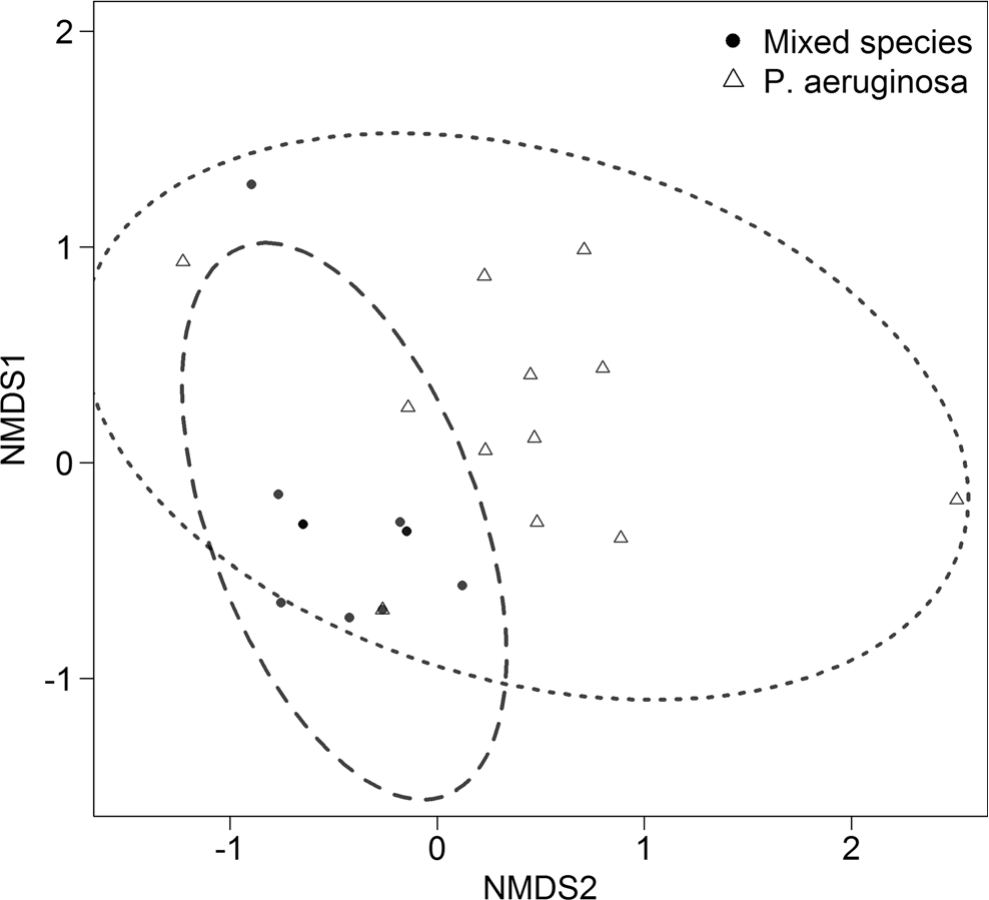
Ecotypes of a single species are more functionally dissimilar than phylogenetically diverse bacterial species. The ability of each isolate to utilise a range of carbon sources was used to determine that the different species (*black circle*) were more functionally similar than the *P. aeruginosa* ecotypes (*white triangle*) using Jaccard’s dissimilarity based on a binary presence/absence matrix. *Dotted* and *dashed lines* represent the 95% confidence interval around the mean distance within the group

Differences were found between the effects of composition (Table 1) in the ecotype and mixed-species experiments; composition did not significantly affect activity in mixed-species assemblages (*F*_11,55_ = 1.10, *P* = 0.38); however, respiration was significantly affected by the different ecotype compositions (*F*_11,55_ = 1.93, *P* = 0.05). To address why composition was important to the variation within the data, each isolate was tested using Biolog EcoPlates™ (Table S2) to assess their ability to utilise specific compounds as single sources of nutrients (analysed as a binary matrix). These results (Fig. 2) indicated that there were significant differences (ANOSIM *R* = 0.345, *P* = 0.01, 99 permutations) between the *P. aeruginosa* ecotypes (mean distance = 0.72 ± 0.17) and the mixed-species (0.44 ± 0.25). This suggested that the significant differences found for the *P. aeruginosa* ecotypes could be due to niche radiation. With the potential to radiate variation in niche usage within a given population, this is a plausible mechanism to explain the observed community dominance of *P. aeruginosa* during late-stage disease [4, 11]. It is interesting, however, that the combining ecotypes did not manage to effect activity as observed when mixing species. This was despite their apparent individual differences, as demonstrated by the carbon utilisation profiles, and the significant effect of composition on the activity values. The rationale for this cannot be elucidated in this study; however, we postulate that due to there being only one type of carbon source present in the media, the ecotypes may not have had the opportunity to interact and increase the overall activity. Further study is required to test our hypothesis.

In conclusion, we have shown that more mixed-species communities display more synergistic interactions, in terms of increased respiration, compared with assemblages of ecotypes from a single species. The differences between the mechanisms (species-species interactions vs. niche diversification) by which variation in the data arose illustrate different ways bacteria can affect overall community function. This study demonstrates the importance of including differing mechanisms of community dynamics when considering biodiversity-ecosystem function experiments; not only are interactions vital for the overyielding of mixed-species assemblages, but strain variation plays an interesting role within community ecology and should be accounted for in ecological analysis of BEF experiments and wider bacterial communities in nature.

## Electronic Supplementary Material

ESM 1 (PDF 185 kb)

Table S2 (DOCX 26 kb)
