# Supplementary material for: Contributions of Composition and Interactions to Bacterial Respiration Are Reliant on the Phylogenetic Similarity of the Measured Community
Source: Microb Ecol. 2017 Apr 27;74(3):757–60. doi: 10.1007/s00248-017-0982-2 (PMC5579169; doi:10.1007/s00248-017-0982-2)
Supplement: Supplementary file 1 — (PDF 185 kb) [file 248_2017_982_MOESM1_ESM.pdf]

Supplementary Materials

Journal: Microbial Ecology – NOTES

Title: Contributions of composition and interactions to bacterial respiration are reliant on the phylogenetic similarity of the measured community

Authors: Damian W. Rivett1†, Andrew K. Lilley[1], Gary J. Connett[2], Mary P. Carroll2, Julian P. Legg[2], Kenneth D. Bruce[1].

Affiliations: 1. Institute of Pharmaceutical Science, Franklin-Wilkins Building, King's College London, UK. 2. UK National Institute for Health Research, Southampton Respiratory Biomedical Research Unit, University Hospital Southampton NHS Foundation Trust, Southampton UK. †Current address: Division of Ecology & Evolution, Imperial College London, Silwood Park campus, Ascot, UK.

Corresponding author: Andrew K. Lilley, Institute of Pharmaceutical Science, Franklin-Wilkins Building, King's College London, UK. Email: andy.lilley@kcl.ac.uk Tel: 020 7848 4670

Table S1 Taxonomic identities of the bacterial isolates that were used to create the multispecies communities.

Taxonomic identifier
Acinetobacter baumannii
Achromobacter xylosoxidans
Burkholderia cepacia
Enterococcus faecium
Pseudomonas aeruginosa
Serratia marcescens
Staphylococcus aureus
Staphylococcus haemolyticus
Stenotrophomonas maltophilia
Streptococcus mitis
Streptococcus pneumoniae
Streptococcus sanguinis

Table S2 Binary utilisation profiles, for all the isolates used in this study, of the single carbon source present in the EcoPlateTM system. A positive result ("+") indicates a value greater than an OD600 = 0.06 above the bacterial respiration in negative control (water).

See Table S2.

Supplementary Methods:

Profiling of bacterial isolates

Bacterial isolates used in this study were isolated directly from expectorated sputa, after treatment with an equal volume of Sputasol (Oxoid Ltd., Basingstoke, UK), on three solid bacterial growth media; Mueller-Hinton (MH), Blood, and *Pseudomonas* selective agar with C-N supplement added (all purchased from Oxoid Ltd.) after 36 hours of aerobic incubation at 33°C shaken at 150 rpm. Colonies were selected at random and subsequent pure cultures were archived at -80°C (Protect™ tubes, Technical Service Consultants Ltd, Heywood, UK) until use. Identification of the isolates was achieved by single extension sequences (performed by Macrogen Ltd., Amsterdam, Netherlands) of 16S rRNA genes and comparison with GenBank reference sequences. Genomic DNA was extracted using a phenol: chloroform extraction protocol with the subsequent PCR amplification [15] of the 16S rRNA gene using the universal primer set 8f (5'-AGA GTT TGA TCC TGG CTC AG-3') and pH' (5'-AAG GAG GTG ATC CAG CCG CA-3'). Composition of experimental microcosms (described below) using was determined at the end of the experiment using Terminal-Restriction Fragment (T-RF) profiling as previously described [15]. If two isolates could not be distinguished the relative abundance was shared equally between them [10]. Carbon utilisation profiles were measured using an EcoPlate™ (Biolog, Hayward, CA, USA), incubated at 37°C with optical density at a wavelength of 590 (OD_{590}) recorded using a SpectraMax 190 spectrophotometer (Molecular Devices, Sunnyvale, CA, USA) after 24 hours post-inoculation. Readings were converted to a binary matrix (0, 1) for analysis.

Assembly of bacterial microcosms

Archived isolates (n = 23) were inoculated into MH broth (Oxoid Ltd.) and incubated at 37°C with shaking at 110 rpm overnight, which was subsequently split into two replicate cultures. Bacterial suspensions were washed twice, resuspended, and diluted to an OD_{600} of 0.1 using 1x M9 salts (Sigma-Aldrich, Gillingham, UK). Mixtures of bacteria were created prior to inoculation with the concentration of cells in each microcosm kept constant at an equivalent OD_{600} = 0.02 (~$1x10^5$ total

colony forming units) regardless of the number of isolates present; each isolate was inoculated at an OD_{600} equivalent to an OD_{600} of 0.02 divided between the number of isolates within a given microcosm.

The microcosms were assembled as described by the Random Partitions Design [14] where, within each set of species combinations, a total of 28 microcosms were created containing either 1, 2, 3, 4, 6 or 12 isolates, with each present once at each richness level. Two pools of isolates were used in this study, one using a mixture of bacterial species and the other using 12 phylogenetically indistinguishable *P. aeruginosa* isolates (one *P. aeruginosa* isolate was used in both pools). The medium used in this study was M9 salts supplemented with 30 mg ml^{-1} Tryptone water (Oxoid Ltd.).

Measurement of CO_2 production

CO_2 production measurements were performed using an adaptation of the MicroResp™ Soil Respiration System (Macaulay Scientific Consulting Ltd., Aberdeen, UK). The concentrations of the reagents in the Cresol Red indicator plate were: deionised water-1% (w/v) agarose (Bioline, London, UK), 150 mM KCl (Sigma-Aldrich), 5 mM $NaHCO_3$ (Sigma-Aldrich) and 12.5 µg/ml Cresol Red (VWR International Ltd., Lutterworth, UK). All indicator plate absorbance readings (λ = 572 nm) were taken using a Spectramax 190 plate spectrophotometer (Molecular Devices,LLC., CA, USA). The calibration of the indicator system was undertaken by exposing the indicator to differing CO_2 levels and a calibration curve of absorbance against $\%CO_2$ was fitted (r^2 = 0.928) to a linear model with log-log transformed variables with these values converted to mass (µg) of CO_2 produced by the microcosm over a 24 hour period ($\mu gCO_2\ d^{-1}$).

Statistical analyses

These data were analysed using a general linear model adapted from Bell *et al.* [14] applying the Random Partitions Design framework. This general linear model included seven explanatory variables

(plus random error, *e*); $y = \alpha + \beta_{LR}x_{LR} + (\sum_{1}^{12} \beta_C x_C) + \beta_I x_I + \beta_{PSP}x_{PSP} + \beta_{MV}x_{MV} + e$, where, α is the model intercept, β is the linear model coefficient and x is the respective coding assigned to each variable; *LR* represents the number of isolates in a microcosm and *C* is the composition of species in a microcosm, with *I* representing the interactions between isolates at a given richness level. *PSP* (partitioned species pool) and *MV* (microcosm variance) are statistical variables required for the analysis as denominators for the F-ratio [14]. ANOVA was applied, with the appropriate F-denominator, to determine whether any of these variables had a significant effect on the total productivity. This general linear model was applied after confirmation of the assumptions of normality and variance homogeneity for the full model. Predicted values were calculated as previously described [10]; $P_{predicted} = \sum_{1}^{N} (ra_i M_i)$, where *N* is the number of isolates in the microcosm, ra_i is the relative abundance of the *i*th isolate in the microcosm (this value was 1 divided by the level of *I* to represent the isolates being equivalent) and M_i is the functioning observed for the *i*th isolate in monoculture. Distance measures between the isolates, based upon binary carbon source utilisation profiles, were calculated using Jaccard's distance in the *R* package *vegan* v2.3-4 (*https://cran.r-project.org/package=vegan).* Significant (where $\alpha < 0.05$) differences between treatments were analysed using both non-parametric tests and parametric statistics with the type of test used stated in the text. For all parametric tests, the data was assessed visually to ensure conformation to the necessary assumptions. All statistical analysis and visualisations were performed in the *R* environment v2.13.0 (https://www.r-project.org).
