# Supplementary material for: Contributions of Composition and Interactions to Bacterial Respiration Are Reliant on the Phylogenetic Similarity of the Measured Community
Source: Microb Ecol. 2017 Apr 27;74(3):757–60. doi: 10.1007/s00248-017-0982-2 (PMC5579169; doi:10.1007/s00248-017-0982-2)
Supplement: Supplementary file 2 — (DOCX 26 kb) [file 248_2017_982_MOESM2_ESM.docx]

| **Class and type of single carbon substrate*** | | **Isolate** | | | | | | | | | | | | **Ecotype** | | | | | | | | | | |
| --- | --- | --- | --- | --- | --- | --- | --- | --- | --- | --- | --- | --- | --- | --- | --- | --- | --- | --- | --- | --- | --- | --- | --- | --- |
|  |  | *P. aeruginosa* | *S. maltophilia* | *B. cepacia* | *A. xylosoxidans* | *A. baumanii* | *E. faecium* | *S.marcescens* | *S. aureus* | *S. haemolyticus* | *S. mitis* | *S. pneumoniae* | *S. sanguinis* | *P. aeruginosa2* | *P. aeruginosa3* | *P. aeruginosa4* | *P. aeruginosa5* | *P. aeruginosa6* | *P. aeruginosa7* | *P. aeruginosa8* | *P. aeruginosa9* | *P. aeruginosa10* | *P. aeruginosa11* | *P. aeruginosa12* |
| Amine | Phenlethylamine | + | + | + | + | + | + | + | + | + | + | + | + |  |  |  | + |  |  |  |  |  |  | + |
|  | Putrescine | + | + | + | + | + | + | + | + | + | + |  | + |  |  | + | + |  | + |  |  | + | + | + |
| Amino acids | L-arginine | + | + | + | + | + | + | + | + | + | + |  | + |  | + | + |  | + | + |  |  | + |  | + |
|  | L-asparagine |  |  |  |  |  |  |  |  |  |  |  |  |  | + |  |  |  |  |  |  |  | + | + |
|  | L-phenylalanine |  |  |  |  |  |  |  |  |  |  |  |  |  | + |  |  | + | + |  |  | + | + | + |
|  | L-serine | + | + | + | + | + | + | + | + | + | + |  | + |  |  |  |  |  |  |  |  |  |  |  |
|  | L-threonine |  |  |  |  |  |  |  |  |  |  |  |  |  |  |  | + |  |  |  |  |  |  |  |
|  | Glycyl-l-glutamic acid |  |  |  |  |  |  |  |  |  |  |  |  |  |  | + | + |  | + |  | + |  |  | + |
| Carbohydrates | D-cellobiose |  |  |  | + | + | + |  |  |  | + |  | + |  |  |  |  |  |  |  |  |  |  |  |
|  | Β-methyl-D-glucoside |  |  |  |  |  |  |  |  |  |  |  |  |  |  |  |  | + |  |  |  |  |  |  |
|  | D-xylose | + | + | + | + | + | + | + | + | + | + |  | + | + |  | + | + | + | + |  | + | + | + | + |
|  | I-erythritol |  |  |  |  |  |  |  |  |  |  |  |  |  |  |  | + |  |  |  |  |  | + | + |
|  | D-mannitol |  |  |  |  |  |  |  |  |  |  |  |  |  | + | + |  |  |  |  |  |  |  |  |
|  | N-acetyl-D-glucosamine |  |  |  |  |  |  |  |  |  |  |  |  |  | + |  | + |  | + |  |  | + | + |  |
| Carboxylic acids | D-galactonic acid γ-lactone |  |  |  |  |  |  |  |  |  |  |  |  |  |  |  |  |  |  |  |  | + |  |  |
|  | D-galacturonic acid |  |  |  |  |  |  |  |  |  |  |  |  |  | + |  |  |  |  |  |  |  |  |  |
|  | 2-hydroxy benzoic acid |  |  |  | + | + | + |  |  |  |  |  | + |  | + | + | + | + | + |  | + | + | + | + |
|  | 4-hydroxy benzoic acid |  | + | + |  | + | + |  | + | + |  | + | + | + | + | + | + | + | + |  | + | + | + | + |
|  | Γ-hydroxybutyric acid | + |  |  |  |  |  |  |  |  |  |  |  |  |  |  | + | + |  | + | + |  | + | + |
|  | Itaconic acid |  |  |  | + | + | + |  |  |  | + | + | + |  | + | + | + |  | + |  |  | + |  | + |
|  | Α-ketobutyric acid |  |  |  | + |  |  |  |  |  |  |  |  |  |  |  | + |  | + |  | + | + |  | + |
|  | D-malic acid |  |  |  | + | + | + |  |  |  | + |  | + |  |  |  | + |  |  |  |  | + |  |  |
| Ester | Pyruvic acid methyl ester | + |  |  | + | + | + |  |  |  |  |  | + |  | + |  |  |  | + |  |  | + | + | + |
| Phosphorylates | Glucose-1-phosphate |  |  |  | + | + | + |  |  |  | + |  | + |  |  |  | + |  | + |  | + |  | + |  |
|  | D,L-α-glycerol phosphate | + | + | + | + | + |  | + | + |  | + |  | + |  |  | + | + |  | + |  | + |  |  | + |
| Polymers | Tween 40 |  |  |  |  |  |  |  |  |  |  |  |  |  | + |  |  |  | + |  |  | + | + | + |
|  | Tween 80 | + | + | + |  | + | + | + | + | + | + |  | + |  |  |  |  |  |  |  |  |  |  | + |
|  | Α-cyclodextrin |  |  |  |  |  |  |  |  |  |  |  |  |  |  |  |  |  | + |  |  |  |  |  |
|  | Glycogen |  |  |  | + | + | + |  |  |  | + |  | + |  |  |  |  |  |  |  | + |  |  |  |
|  | Total | 9 | 9 | 8 | 8 | 14 | 15 | 14 | 7 | 8 | 7 | 12 | 3 | 2 | 2 | 11 | 9 | 15 | 7 | 15 | 1 | 9 | 13 | 12 |
